# Kinetic Pathways to Gelation and Effects of Flow-Induced Structuring in Depletion Gels

**DOI:** 10.1021/acs.iecr.4c03873

**Published:** 2025-02-12

**Authors:** Gabriele Colombo, Pierre Lehéricey, Florence J. Müller, Madhu V. Majji, Hanumantha Rao Vutukuri, James W. Swan, Jan Vermant

**Affiliations:** † Department of Materials, ETH Zürich, CH-8093 Zürich, Switzerland; ‡ Department of Chemical Engineering, Massachusetts Institute of Technology, Cambridge, Massachusetts 02139, United States; § Active Soft Matter and Bio-inspired Materials Lab, Faculty of Science and Technology, MESA+ Institute, University of Twente, 7500 AE Enschede, The Netherlands

## Abstract

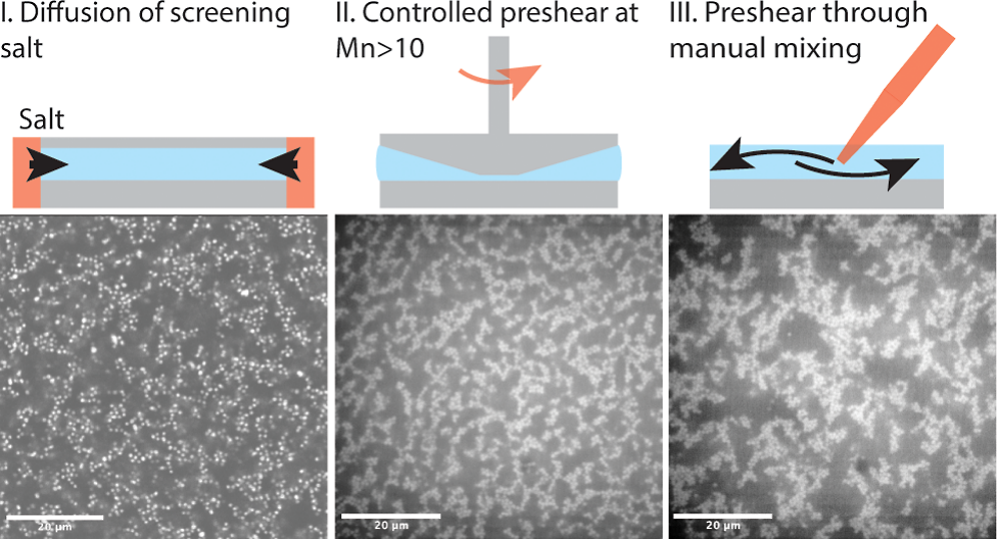

The kinetic pathways to gelation and the effects of flow-induced restructuring are studied here in depletion flocculated gels with short-ranged attractions, both experimentally and using computer simulations. In the experiments, we first carefully diffuse a screening organic salt to destabilize colloid–polymer mixtures and form a gel. We hence avoid flow history effects, typical of traditional mixing protocols. The initial gelation phases are then accessible and observed by time-resolved confocal microscopy. These insights show that quiescent gelation reduces heterogeneity and strand size with increasing attraction strength, with deeper quenches leading to earlier arrest. These findings are consistent with the simulations which include long-range hydrodynamic interactions. We then compare these results with gels formed by high-rate preshear followed by cessation of colloid–polymer–salt mixtures. The obtained microstructures do not seem in this case to depend on depletant concentration. Indeed, confocal images reveal that shear flow significantly impacts gel structure, from fluidization at high shear rates to dense heterogeneous aggregates formation at lower rates. We especially show how the heterogeneity is controlled by the strength of the flow relative to the attraction forces between the colloids. This study highlights the subtleties behind the preparation protocols of colloidal gels. In particular, it shows that differences in kinetic aggregation pathways can overshadow attraction effects, such as those caused by varying flow conditions during mixing at different attraction strengths. These insights provide a framework for understanding gelation kinetics and optimizing structural reproducibility in colloidal gel experiments.

## Introduction

The stability of colloidal suspensions is known to depend on the interplay between repulsive and attractive interparticle forces.^1^ When the latter dominate the interaction potential, aggregation of particles can lead to sample spanning networks, even at low particle loadings.^2^ The elastic properties of the resultant colloidal gels are crucial for their application in consumer products and technological applications.^3−7^ Another key characteristic of these materials for their applications is their elasto-visco-plastic nature. Indeed, they can transition from solid-like to liquid-like behavior upon exceeding a critical stress range and vice versa.^8,9^ They also display a time dependent viscosity.^10^ These characteristics are respectively termed yielding and thixotropy. Both of these features find their origins in the evolution of the microstructure. While yielding is related to a breakdown of the colloidal network, thixotropy is associated with the recovery of this latter. These effects emphasize the consequences of the state of the microstructure on the bulk rheological properties of colloidal gels. They are even more pronounced at low volume fractions when the network is sparse.^11^

There are several different pathways to create the gel structure, ranging from (i) purely kinetic ones, related to random percolation^9,12−15^ over (ii) percolation of particle clusters^16,17^ to (iii) thermodynamically driven instabilities for even stronger attractive systems resulting in arrested phase separation with clear spinodal structures.^18,19^ Much insight has come from simulations, where the routes to colloidal gelation can be explored readily by varying the lifetimes of the colloidal bonds^19^ relative to the quenching speeds. Moreover, recent advancements in computational efficiency allowed researchers to demonstrate how hydrodynamic interactions alter gelation by changing percolation kinetics and lowering the gelation line.^20−22^ From an experimental point of view, different research groups have reported discrepancies in gel structures obtained from similar formulations.^15,18,23,24^ These differences are particularly evident in the gels strand size, the occurrence of dynamic and structural heterogeneity, and their dependence on interparticle attraction. Flow history during or immediately after gelation might explain these variations. Even small flows during this process appear to tune the gel’s microstructure, making these materials highly sensitive and challenging to manipulate.^25−27^

Moderate attraction strengths between particles, on the order of a few times the thermal energy (*k*_B_*T*), lead to weak physical gels where flow can be expected to have a strong effect on the structural heterogeneity. These effects can be rationalized using the Mason number, which is the ratio of the shearing forces over the attractive forces in the gel. It can be written as  with η the viscosity of the solvent, γ̇ the shear rate, *a* the particle radius and *U* the attractive potential strength. Strong flows (*Mn* > 15) fully break up the structure, which leads to more homogeneous and stronger gels being formed after shear cessation. The same operation at low flow rates creates largely heterogeneous weaker gels with reduced elasticity.^25^ This can be rationalized by the interplay between interparticle attraction and hydrodynamic stresses which controls the “life and death”^28^ of colloidal bonds^28,29^ and the presence of aging.^30^ Flow preshear protocols have hence been suggested as a way to obtain reproducible results. Nevertheless, the abundance of preshear protocols which can include large amplitudes oscillatory shear, shear rate step-downs or shear rate reversals have led to discussions questioning which protocol was the best to achieve a reproducible initial gel structure.^10,25−27,31,31−34^ However, interestingly, the effects of flow during gel preparation and mixing are often not considered.

Some of the most studied model weak gels use depletion forces, which involve an attraction between particles created by the entropic effects of adding a nonadsorbing polymer to the gel’s suspending medium.^35^ By adjusting the polymer concentration and size, the interaction strength and range can be finely controlled. Model systems suitable for such microscopy and rheology studies, using brightly fluorescent PMMA particles with a grafted brush layer of polyhydroxystearic acid^36−38^ use a refractive-index-matched and density-matched mixture of cyclobromohexane and *cis*-decaline with polystyrene as a depletant.^39^ Contrary to thermoreversible gels, where flocculation can be induced with great control by variations of temperature,^40,41^ attractive interactions are introduced by mixing the different components. This complicates the study of the initial phases of gel formation and may introduce flow history effects. These effects depend on the specifics of the mixing and dispensing protocol, which is often overlooked. Nevertheless, such protocols are likely to influence systems with strongly attractive forces.

Detailed confocal microscopy studies by Lu and co-workers^18^ showed that the formation of depletion gels at volume fractions of about ϕ = 0.2 showed clear arrested spinodal decomposition. A thermodynamic instability causes density fluctuations, leading to space-spanning clusters that dynamically arrest to form a gel. Dibble and Solomon suggested that dynamic heterogeneity adds a layer of complexity in this mechanism: as the short-range attractive interaction strength increases, structurally more heterogeneous clusters and dynamic immobilization were observed. This was shown respectively by number-density fluctuations and single-particle mean-squared displacement.^23^ An additional source of complexity in these PMMA depletion gels arises from the presence of repulsive electrostatic interactions. They are indeed known to play an important role in dispersing media with low dielectric constant.^42^ Recently, controlled screening of the electrostatic repulsion using organic salts which are left to diffuse through a membrane^43^ resulted in a controlled screening of the repulsive interactions.

In the present work, we built on this approach to systematically investigate the effects of interparticle attraction strength and steady state shear flow on the microstructure of a model depletion gel at intermediate volume fraction (ϕ = 0.2). Particular care was taken to ensure reproducible initial structures, by using either a diffusion step of screening salt into a charge stabilized colloid–polymer mixture, or well-defined preshear protocols. By applying these procedures, we observed the early stages of gel formation across a wide range of depletant concentrations, from shallow to deep quenches with high attraction strengths. Early arrest and fine particle strands emerged in the latter case. Using a recently developed rheoconfocal setup,^44^ we live-imaged the microstructure under steady flow at a stagnation plane to study shear-induced rearrangements. Variations in gel heterogeneity, driven by the shear rate, were dramatic and far exceeded the influence of depletant concentration. The results are compared to detailed simulations that incorporate far-field hydrodynamics under both quiescent conditions and linear shear flow. The goal of this manuscript is to elucidate the influence of kinetic pathways during gelation on the microstructures observed in order to have robust protocols for reproducible experiments, which enable a comparison with simulations or theory.

## Materials and Methods

### Model Depletion Colloidal Gel

The colloidal particles used in the gel are made of poly(methyl methacrylate) and are sterically stabilized by a grafted thin layer of polyhydroxystearic acid (PMMA-*g*-PHSA). Detailed protocols on the preparation of the stabilizer as well as the particle synthesis are described elsewhere.^36,38,45^ In short, functionalized fluorescent rhodamine B is copolymerized to PMMA during the particle synthesis. The stabilizer was then grafted to the obtained particles in a standard locking step. Particles obtained had a diameter 2*a* = 1.1 μm and a polydispersity index lower than 5%, assessed by DLS. Particles were suspended in a mixture of 80 wt % cyclobromohexane (CHB, Acros Organics) and *cis*-decaline (Tokyo Chemical Industry) to match their density and refractive index.^46^ In this suspending medium, the particles swelled to a final particle diameter of 2*a* = 1.2 μm. Depletion gels were obtained by mixing a stock particle suspension with solutions of polystyrene with a molecular weight *M*_w_ = 925 kDa (PS, Polymer Standards Service GmbH), nonadsorbing to the PMMA-*g*-PHSA particles. The PS radius of gyration in the solvent mixture was determined to be 41 ± 4 nm.^23^ The resulting PS overlap concentration was therefore estimated to be of c* = 5.4 mg/mL. Model depletion gels reaching a final volume fraction ϕ = 0.2 were prepared with a range of PS concentrations *c*/c* = 0.25–1.25, which correspond to depletion attraction strengths at contact of 5–27 *k*_B_*T*, as estimated with the Asakura–Oosawa interaction potential^47,48^ (see Supporting Information Figures S1 and S2). Even if this model assumes a low *c*/c* and could be refined at high *c*/c* with more advanced models,^49,50^ it still constitutes a reasonable approximation at higher depletant concentrations used in other studies.^23,26^ The attractive interaction potential is short-ranged, with a polymer to colloid size ratio of ξ = *R*_g_/*a* = 0.07.

However, the PMMA-*g*-PHSA particles are known to acquire a positive charge when suspended in CHB and *cis*-decaline.^51^ The particles charge due to the release of hydrobromic acid (HBr) following the decomposition of CHB. HBr hence behaves as a weak acid and shows a finite dissociation, so that H^+^ ions are present in solution. The subsequent adsorption of these protons on the stabilizing PHSA layer of the particles was posited to be the cause of the positive particle charge.^42,52^ The organic salt, usually tetrabutylammonium chloride or bromide (TBAC or TBAB), effectively screens the electrostatic interaction between charged particles. As a consequence, the range and strength of electrostatic repulsion decrease as the ionic strength of the solvent mixture increases.

In a first approximation, the interaction potential is better estimated by extending the Asakura–Oosawa model as described in eq 1. This extension assumes the model remains valid despite a small amount of charges that may slightly alter the conformation of the depletant. It also includes an additional repulsive term, represented by a Yukawa potential, to account for screened Coulomb interactions
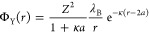
1where *Z* is the number of elementary charges per particle, *r* the center-to-center interparticle separation and *a* is the particle radius. The Bjerrum length is the distance at which the Coulomb potential between two colloids equals the thermal energy: λ_B_ = *e*^2^/4πε_r_ε_0_*k*_B_*T*. The Debye length highlighted in eq 2 characterizes the range of the screened electrostatic interaction
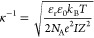
2where ε_r_ is the dielectric, ε_0_ is the vacuum permittivity, *k*_B_ is the Boltzmann constant, *T* is the temperature, *N*_A_ is Avogadro’s number, *e* is the elementary charge, and *I* is the ionic strength. It is to be noted that the barrier to gelation in this summed potential depends on the depletant concentration (see Figure S1).

### Conductivity Measurements to Monitor PMMA-*g*-PHSA Protonation

The degree of dissociation of HBr and TBAC in CHB is controlled and quantified by conductivity measurements. In order to reliably measure the conductivity in low permittivity media, down to the pS–nS/cm range, a custom cell consisting of two round stainless steel electrodes with a diameter of 40 mm was used. A polyoxymethylene ring spacer with a thickness of either 1 or 3 mm was clamped between the electrodes, so that the sample of interest could be injected in the resulting gap with a syringe. The active area of liquid in the gap was *A* = 7.07 cm^2^. The sample was injected while keeping the cell vertical, filling from its bottom to avoid the formation of bubbles. The impedance was measured with a Biologic SP200 impedance analyzer, by applying a sinusoidal voltage of 300 mV in a frequency range 400 kHz to 400 mHz. From the measured resistance *R*, the real part of the impedance, the conductivity was calculated as .

CHB purchased from different suppliers showed significant differences in the measured conductivities (Table 1), indicating varying amounts of dissolved HBr. In some cases, a yellowish color was also apparent, indicative of the presence of Br_2_. Since the solution conductivity stems from the product of a decomposition reaction, it was found to vary depending on storage time as well. As reported previously,^53^ various cleaning protocols can be applied to remove traces of polar contaminants such as HBr and water. The simplest and most effective method consists in contacting the CHB with activated alumina (MP Biomedicals alumina B, activity level 1). Stirring 20 mL of sample overnight with 10 mL of such a basic alumina powder was sufficient to remove most of the HBr and drop the conductivity to 50 pS/cm, eliminating the yellow color as well. In order to introduce a known amount of HBr and obtain consistent charging of the particles, gaseous HBr was bubbled through a previously cleaned CHB sample. Using a flow rate of 40 mL/min of 50% v/v HBr and helium, 15 min of bubbling time were sufficient to saturate 25 mL of CHB, leading to a much larger conductivity than found in the commercial solvent batches. Immediately diluting the saturated sample with clean CHB yielded well-controlled conductivities. Longer storage of saturated CHB resulted in a yellow coloring within 1 day, and eventually a black sediment formed.

**Table 1:** Appearance and Conductivity σ of CHB from Different Suppliers for Different Treatments^a^

supplier	treatment	σ [nS/cm]	appearance
Sigma-Aldrich	as received	0.9	colorless
Sigma-Aldrich	stored 2 years	2.1	colorless
Alfa Aesar	as received	6.4	colorless
Acros Organics	as received	3.1	yellowish
Acros Organics	alumina cleaned	0.05	colorless
Acros Organics	cleaned, saturated HBr	146.3	colorless
Acros Organics	cleaned, saturated HBr, 50× diluted	12.2	colorless

a A large variation in dissolved HBr was found, potentially leading to a range of particle charges.

The conductivity of the suspending medium was used to calculate the Debye length from the ionic strength *I*, by applying Walden’s rule as in Royall et al.^42^ The ion valency for TBAC is *z* = 1. The sum of attractive and repulsive contributions yielded the interaction potentials shown in the Supporting Information Figure S1b. A kinetic barrier emerged for the charge-stabilized colloid–polymer mixtures in the Supporting Information Figure S1a, resulting from electrostatic repulsion (without the addition of the screening salt TBAC). The Debye length in these conditions was κ^–1^ = 150 nm. Upon addition of the screening salt, the screening length decreased to κ^–1^ = 55 nm, resulting in a purely attractive interaction potential. The number of charges *Z* = 175 was estimated from the zeta potential of approximately 20 mV measured by dynamic light scattering in dilute conditions (ϕ = 0.0003) using a Zetasizer from Malvern. This value represents a rough estimate, as the adsorption equilibrium of ions on the particles was demonstrated to depend nonlinearly on the suspension volume fraction.^52,54^ Additionally, we verified that the particle charge decreases and reverses sign upon introduction of TBAC screening salt.^42^ We then used *Z* = 100 to estimate the potential.

### Confocal and Rheoconfocal Microscopy

Flow experiments were performed using an in-house developed, ultrafast, rheoconfocal shear setup.^44,55^ A Twindrive-ready Anton Paar MCR 502 WESP rheometer (with exposed support plate for optical access) was coupled to a fast scanning, instant structured illumination (iSIM) confocal microscope from VisiTech International. A second Twindrive motor was integrated into a custom-made setup, allowing counter-rotation of the lower transparent glass plate. This counter-rotation effectively moves the zero-velocity plane to any position in the shearing gap, depending on the ratio of upper and lower angular velocity. As a result, colloids can be imaged under steady shear with shear rates up to 100 s^–1^ at a stagnation plane for extended periods. The shear rate remains constant over the gap height, validated by particle image velocimetry experiments on a stable suspension described elsewhere.^44^

100 μL of the sample were dispensed on the coverslip, and the upper geometry was immediately lowered to the measuring position. A cone–plate geometry with a cone angle of 6° and a diameter of 15 mm was used. The relatively large cone angle allows the exploration of a wide range of shearing gaps with small radial displacements of the imaging axis from the center of rotation.^44^ Flow experiments at the stagnation plane were performed while imaging at a position 2 mm off the center of rotation, leading to a shearing gap of 210 μm. Confocal images were obtained at a depth of at least 25 μm in the sample.

The iSIM scanner allows fast confocal imaging at improved resolutions (180 nm laterally, 440 nm axially, up to 1000 fps), thanks to a purely optical implementation of the structured illumination concept.^56^ Further increase in resolution by a factor of 1.4 can be obtained by subsequent image deconvolution with the measured point spread function of the imaging system. We exploited the high temporal and spatial resolution of our confocal setup to attain large fields of view while maintaining single particle resolution. The resulting lateral image sizes were 150 × 150 or 100 × 100 μm, obtained using a 60× or a 100× oil immersion objective, respectively (Nikon Plan Apo Lambda, NA = 1.4/1.45).

### Image Analysis

Particle coordinates were obtained from the confocal micrographs using the standard methods of Crocker and Grier.^57^ From this information, we derive structural descriptors of interest to compare relatively the effects of the different kinetic pathways of gelation on the final colloidal structure.

In order to capture the variations of heterogeneity in the microstructure after gelation at a mesoscale level, the number density fluctuations were chosen in line with previous works in colloidal gels.^17,23,58^ Number fluctuations were computed by randomly placing boxes of varying size on the field of view and counting the particles within. The variance in the number of particles in different frames was then calculated for a fixed box size and normalized by the mean number of particles, yielding the number density fluctuations as described in eq 3
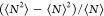
3The counting boxes were placed randomly in 100 different positions uniformly distributed across each frame.

Based on a more local description, Voronoi tessellations were computed to study the temporal development of the microstructure during the gelation, in line with previous works as well.^58,59^ In heterogeneous gels, Voronoi tessellation can capture spatial variations in local microstructure, revealing patterns of cluster formation, and the distribution of voids between network elements and we use the same method here as in these earlier studies. These tessellations were obtained using the built-in functions in Matlab. The distributions of the areas in the tessellation are normalized by the area of the largest cross-section of a particle. Moreover, descriptors at the particle scale, i.e. pair distribution functions and coordination number distributions were obtained using the built-in functions of the OVITO package.^60^ These latter distributions are reported in Supporting Information Figure S6, as this local descriptor is less sensitive to variations in the mesoscale structures that we focus on.

### Simulations

A far-field Stokesian dynamics plugin was used with the HOOMD-blue suite of molecular dynamics simulation software to simulate the colloidal suspensions. The long-range hydrodynamics between monodisperse spheres were accounted for using the Rotne–Prager–Yamakawa tensor with the grand mobility tensor framework, and a positively split Ewald algorithm was employed to compute the Brownian displacements of the colloids.^61^ Lubrication interactions were ignored in this work. The short-range depletion attraction between the colloidal particles described in eq 4 was modeled using the Asakura–Oosawa form
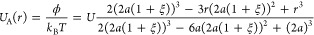
4for 2*a* < *r* < 2(*a* + ξ) where *r* is the center-to-center particle separation, ξ is the range of attraction and *U* is the strength of depletion attraction between colloids at contact.^47,48^ Gels with three different contact depletion strengths *U* = 10.6 *k*_B_*T*, 16.2 *k*_B_*T* and 27.4 *k*_B_*T* were simulated with ξ = 0.0745*a*, which correspond to the experimental realizations. The screened Coulomb repulsive interactions between the colloids were accounted for by the pairwise Yukawa potential for *r* > 2*a*. In addition, a hard-sphere repulsive potential is used when *r* < 2*a* to avoid particle overlap. A configuration of monodisperse hard spheres, generated from a Brownian dynamics simulation in a cubic box corresponding to a volume fraction of ϕ = 0.2, was used as an initial condition. Depletion attraction and electrostatic repulsion were applied on the particles immediately at the beginning of the simulation and kept active for the full simulation duration. For each simulation, 10,684 particles are simulated in a cubic box of size 60.64 to achieve an average colloidal volume fraction of ϕ = 0.2. The simulation is run for a duration of 1200 times the diffusion time scale of the colloidal particle. Data from the last 200 diffusion times is used to calculate the statistically averaged quantities of the gel microstructure. Each simulation was run on a single GPU (GeForce GTX 1080 Ti from NVIDIA) with 33 MHz clock speed and 11,264 MiB memory. Simulation run time was anywhere between 14 and 19 GPU hours depending on the input conditions.

### Gelation Protocols

#### Experiments

In the experiments, two main strategies were followed to ensure reproducible initial gel microstructures. In a first set of experiments, the gelation was investigated in the absence of mixing flows by starting from a stable electrostatically repulsive dispersion and then gradually screening the initial charge by diffusion of the screening salt into the colloid–polymer mixture. Controlling the initial particle’s charge degree through the dissociation of HBr in CHB is done in such manner that the experiment is started from a stable dispersion, before salt is induced. For such quiescent gelation experiments, the PMMA-*g*-PHSA particles were suspended in a mixture of *cis*-decalin and the CHB with a conductivity of 12.2 nS/cm, prepared as described above. Mixing such a stock suspension with a PS solution yielded charge-stabilized colloid–polymer mixtures. These were loaded in coverslip cells, prepared by gluing standard #1.5 coverslips with an UV curing Norland optical adhesive 61. Pieces of a #2 coverslip were used as spacers to obtain a gap of 200 μm. 40 μL of colloid–polymer mixture were then dispensed in the coverslip cell with a micropipette. A solution of 260 μM TBAC in the same density matching solvent mixture, and having the same concentration of PS depletant, was carefully introduced in the cell and contacted with the colloid–polymer mixture. After sealing the cell with the UV glue, the progression of the TBAC diffusion front and the resulting gelation were monitored by confocal imaging. We did not observe any significant diffusiophoretic motion, in line with the report of Tanaka et al.^43^

In a second set of experiments, the gelation was investigated in the presence of mixing flows. TBAC was mixed with the colloid–polymer mixture by 10 s of vortex mixing, reaching a concentration *c*_TBAC_ = 40 μM before loading the samples in the rheometer. In order to break up the aggregates formed after the loading step, whether a controlled preshear protocol at a shear rate γ̇ = 100 s^–1^ followed by either a step-down in shear rate or quiescent structural recovery was carried, whether stirring with a pipet tip after deposition of the gel on the coverslip was performed.

#### Simulations

To understand if the observed change in microstructure between the two sets of experiments is due to preshear or nonuniform salt concentration, we performed simulations with two different protocols. First, we simulated the gel evolution under quiescent conditions with and without preshear while keeping the salt concentration homogeneous in time and space. The second protocol involved simulation of gel evolution under quiescent conditions with no preshear but with two concentration distributions of the salt: (i) uniform across space and time and (ii) slowly varying in time and uniform in space.

## Results

### Quiescent Gelation

Quiescent gelation experiments were carried out by allowing the TBAC screening salt to diffuse into charge-stabilized colloid–polymer mixtures. We note that the time required for the salt ions to diffuse from the entrance of the sample cell to the location where the data was recorded is of the same order of magnitude^43^ (≈10 s) with respect to the Brownian time of the particle (≈6 s). Since the salt diffusion time is an order of magnitude faster than the typical gelation time in our experiments, the gelation process is assumed to be not limited by the salt diffusion.

**Figure 1:**
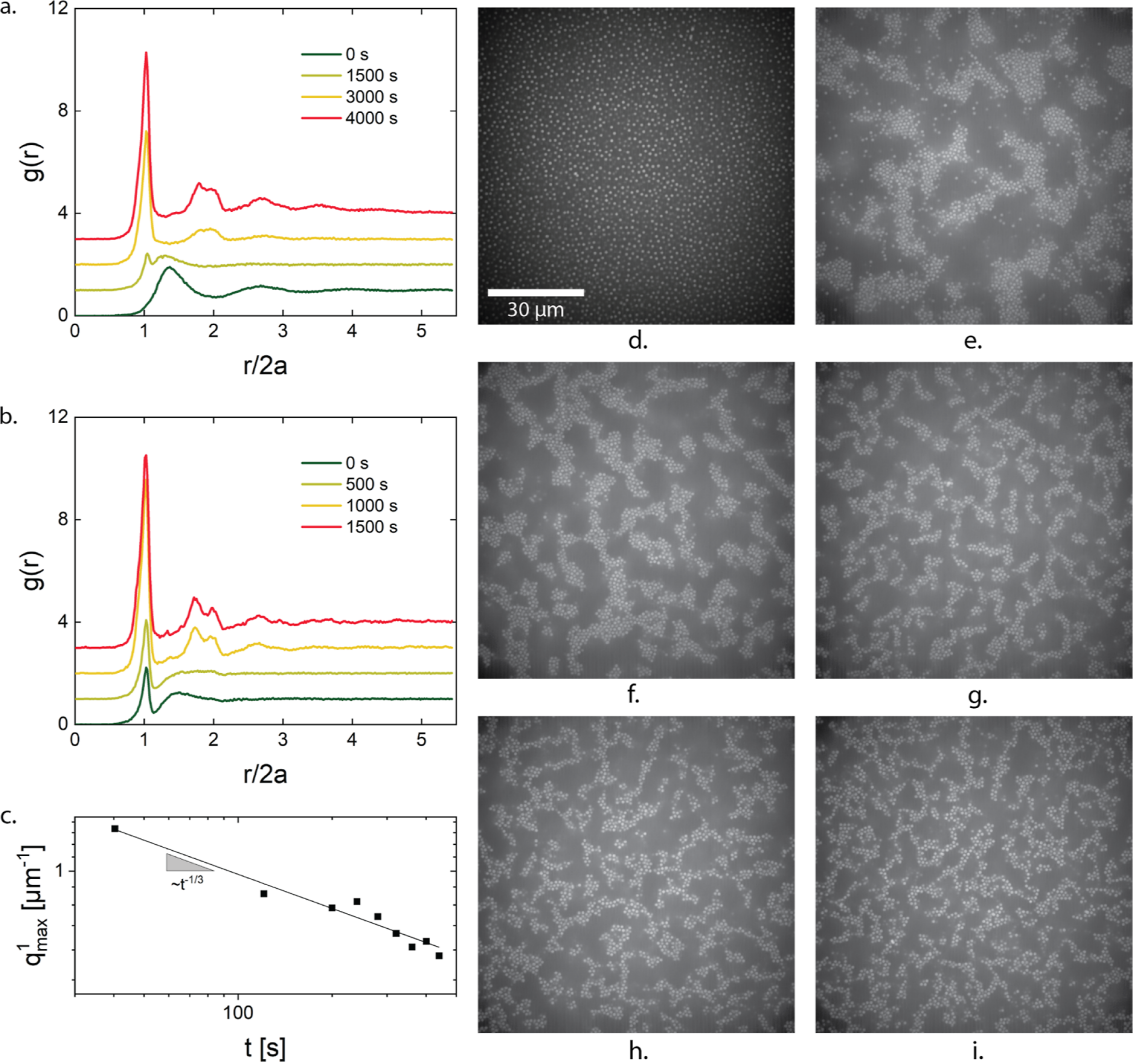
(a,b) Evolution of the pair distribution function *g*(*r*) upon diffusion of the screening salt TBAC, for two interparticle potentials *U* = 5 *k*_B_*T* (a) and *U* = 13.4 *k*_B_*T* and a volume fraction of ϕ = 0.2. (b) The curves are offset by a constant value of 1 for clarity. (c) Temporal evolution of the dominant wave vector of the structure factor for a suspension at *U* = 13.4 *k*_B_*T* and a volume fraction of ϕ = 0.2. The scaling confirms spinodal decomposition (equivalent scattering vector). (d) Representative confocal micrograph of the repulsive particle suspension. (e–i) Representative confocal micrographs of depletion gels obtained by the salt screening quiescent gelation protocol, as a function of the attraction strength: (e) *U* = 5 *k*_B_*T* (f) *U* = 7.8 *k*_B_*T* (g) *U* = 10.6 *k*_B_*T* (h) *U* = 13.4 *k*_B_*T* (i) *U* = 16.2 *k*_B_*T*.

The gel structures resulting from this preparation protocols at different depletant concentrations are shown in Figure 1. Time-lapse movies of the gelation process at different attraction strengths can be found in Movies S1–S3 (S1: *U* = 5 *k*_B_*T*, S2: *U* = 7.8 *k*_B_*T* and S3: *U* = 13.4 *k*_B_*T*). Considering a minimal attraction strength of *U* = 5 *k*_B_*T*, all samples evolved to a sample-spanning gel structure. However, the final microstructures were strikingly distinct, and the time to reach the gel state once the TBAC diffused along the field of view also differed. A monotonous decrease in the strand size and a faster arrest follow from the increase in attraction strength.

The shallowest quench into the gas–liquid coexistence region was realized with a polystyrene (PS) attraction strength of *U* = 5 *k*_B_*T*. The evolution from a stable to an attractive suspension upon diffusion of TBAC is clearly reflected in the pair distribution function *g*(*r*), shown in the Figure 1a at different time points during the diffusion protocol. Initially, a peak was observed at *r*/2*a* ≈ 1.4, followed by broader and shallower ones at *r*/2*a* ≈ 2.7 and 4. As the screening salt diffuses into the colloid–polymer mixture, the long-range organization disappeared and *g*(*r*) became flatter at all ranges, which is expected for hard spheres at modest volume fractions. However, small aggregates started to form during the transition, as signaled by the appearance of a sharp peak at *r*/2*a* = 1.02. The further growth and coalescence of the clusters was well tracked by the pair distribution function, which showed a rise of the first near-contact peak, as well as the appearance of a pronounced second one. The latter displayed a split into two subpeaks, typical of hexagonal close packing. At the lowest attraction strength, the gelation time was significantly longer than the time of diffusion of the screening salt across the field of view. For this attraction strength (*U* = 5 *k*_B_*T*), even in the late stage of network formation, free particles and small clusters are found in the void spaces. This indicates a dynamic particle equilibrium between the colloid-rich phase (strands) and the gas phase (voids). Thus, in this system (most likely on the spinodal, *U* = 5 *k*_B_*T*) gelation proceeds near equilibrium conditions until arrest occurs, most likely forming an attractive glass in the colloid-rich phase.^18,19^

At higher depletant concentrations, gelation proceeds more abruptly upon diffusion of TBAC into the colloid–polymer mixture, as reflected by the *g*(*r*) in Figure 1b for the case *U* = 13.4 *k*_B_*T*. At this attraction strength, few small aggregates are present initially, while further aggregation is prevented by electrostatic repulsion. This reflects a more complex interaction potential, where the repulsive and attractive contributions combine to create a kinetic barrier and a net attractive well at contact (see Supporting Information Figure S1b). The occurrence of stable clusters in systems with short-range attraction and long-range repulsion has been previously demonstrated for e.g. protein solutions.^62^ From this stable initial state, the formation of a sample-spanning network proceeded in less than 500 s (50 times faster than the TBAC diffusion time), as evidenced by the appearance of a clear split peak with maxima at *r*/2*a* = 1.7 and 2, along with a sharp increase in the height of the near-contact peak. Qualitatively consistent with previous results,^18^ at these attraction strengths, large strands form and coarsen over time. The observed formation mechanism follows the framework established by the confocal measurements of Lu et al.,^18^ where gelation is driven by arrested phase separation as is confirmed by the scaling in Figure 1c.

**Figure 2:**
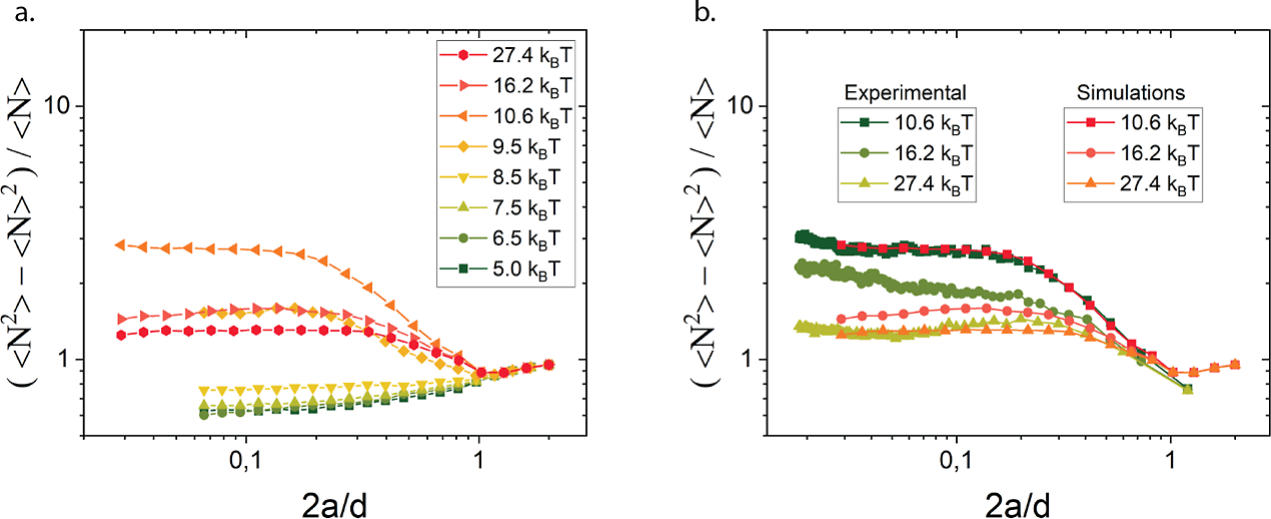
Number density fluctuations as a function of the ratio of particle diameter 2*a* to size of the box *d* in which density fluctuations are computed to highlight and verify gelation boundary. (a) Number density fluctuations predicted by simulations show a gel boundary around *U* = 8.5 *k*_B_*T*. (b) Direct comparison of simulation predictions with experiments results.

The simulations of the number density fluctuations of the structure at steady state predict gelation to occur for ϕ = 0.2 when *U* ≥ 8.5 *k*_B_*T*. Stable clusters started appearing between *U* = 7.5 *k*_B_*T* and *U* = 8.5 *k*_B_*T* above which they were observed to form into system-spanning networks, which can be seen in Figure 2. With further increase in the attraction strength, the microstructure gets more stringy and more homogeneous, as the size of the dense clusters and particle depleted regions diminishes. This resulted in the lowering of the plateau in the number density fluctuation plots. The simulation predictions (at *U* = 10.6 *k*_B_*T*, 16.2 *k*_B_*T*, and 27.4 *k*_B_*T*) agree well with the experiments in which the microstructure snapshot used to compute the number density fluctuation was taken 3 min after diffusion of the salt, when no particle rearrangement was observable. Stable clusters were also observed at *U* = 7 *k*_B_*T*. For *U* = 5 *k*_B_*T* the clusters coexisted with the gas (see Figure 2).

**Figure 3:**
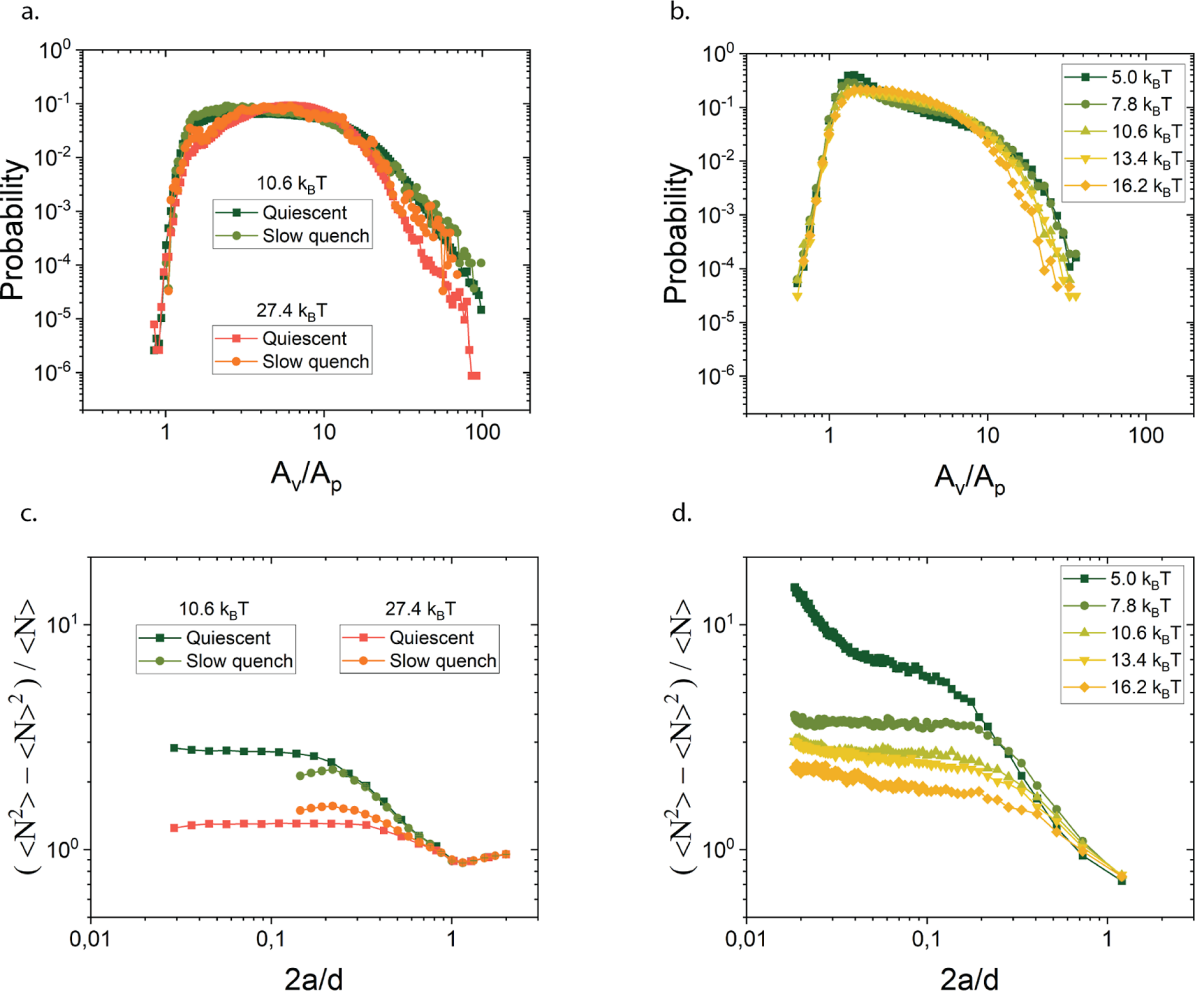
Probability distribution of Voronoi areas (a,b) and number density fluctuations versus box size (normalized by the particle size) (c,d) in simulations (a,c) and experiments (b,d).

**Figure 4:**
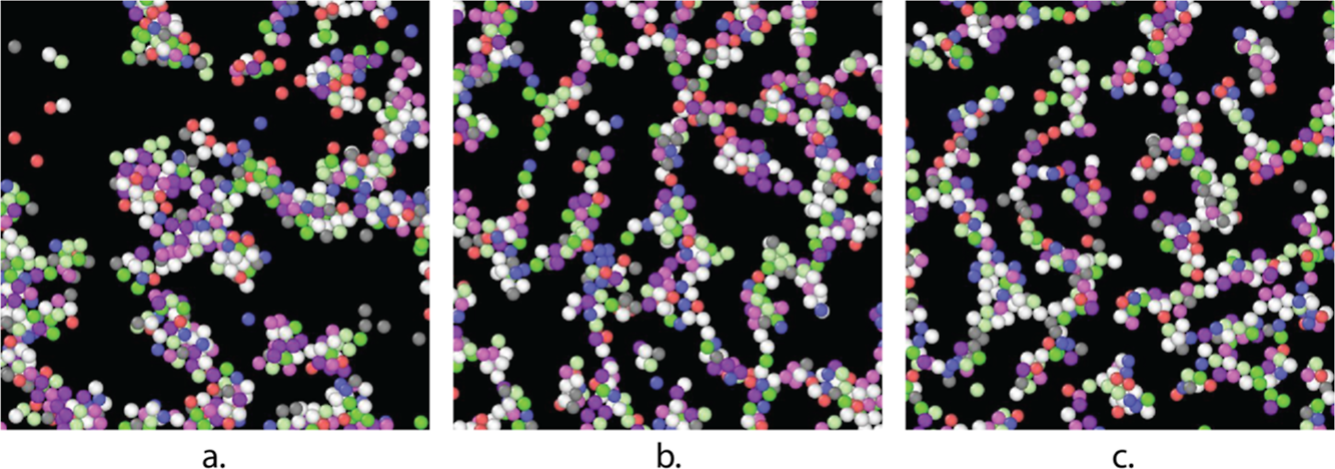
Representative gel microstructure predicted from simulation following an instantaneous quench with (a) *U* = 10.6 *k*_B_*T*, (b) *U* = 16.2 *k*_B_*T*, (c) *U* = 17.4 *k*_B_*T*. The particle color was randomly assigned by the processing software and does not have any significance.

The pair distribution function *g*(*r*) is relatively insensitive to the structural differences at larger length scales. Both the number density fluctuations as a function of box size and the distribution of Voronoi tessellations can be used to provide more insight, for example Figure 3, helps us understand the packing, structural heterogeneity, and the impact of interaction strengths on the overall gel structure. Figure 3 compares the results of simulations and experiments. In the case of shallow quenches, both the experiments and simulations show a peak at small Voronoi areas (*A*_v_/*A*_p_ ∼ 1). This is indicative of interstitial spaces of closely packed particles in the inner regions of the large strands. With increasing attraction strength the distribution broadens up to *A*_v_/*A*_p_ ∼ 100, and develops a shoulder starting at interparticle potentials of *U* = 7.8 *k*_B_*T*. These distributions indicate the presence of large voids (order of 10 to 100 times the area of the individual particles) in the material, with Voronoi cells at the strand edges extending into these empty spaces. The same trend is observed when comparing the experiments with simulations at *U* = 10.6 *k*_B_*T* and *U* = 27.4 *k*_B_*T*, where additionally the effect of quench speed was investigated (see Figure 4).

The number density fluctuations (see Figure 3c,d) reveal a similar picture. A monotonic trend was observed in the number fluctuations as a function of the attraction strength, the structure becoming less compressible (lower plateau value of the number density variations) with increasing stickiness. At the lowest interparticle potentials *U* = 5 *k*_B_*T*, the variations reached an approximately constant value for 2*a*/*d* < 0.09 (of course, realizing that for smaller values of 2*a*/*d* the statistics become poorer), that is for length scales larger than about 10–11 particle diameters. The same qualitative behavior was observed for the other attraction strengths, with a systematic decrease in the magnitude of the number variations with increase in depletant concentration. Additionally, on small length scales, the number fluctuations no longer depend on the depletant concentration suggesting that internal strand structure is similar. The experimental results show a peak at values close to 1, which reflects differences in the local packing, but this minor difference was not further investigated and is probably related to the absence of the near field hydrodynamics in the simulations. For *U* = 7.8 *k*_B_*T* the transition to a plateau in the number fluctuations was found at about 6 particle diameters.

### Structural Heterogeneity: Quench Following Preshear Versus Quiescent Quench

Controlled quenches in the gas–liquid coexistence regions could be obtained by the described salt diffusion protocols and yielded arrested spinodal structures for all attraction strengths. These well controlled quenches can now be compared to those often used in experimental studies where a preshear protocol is used, which is often called “rejuvenation”.^34,63^ Using a rheoconfocal setup, an instantaneous quench following the stopping of flow after a preshear protocol at 100 s^–1^, corresponding to a Péclet number . This protocol was applied to samples already containing the screening salt TBAC uniformly distributed, at a concentration *c*_T_ = 40 μM. In these conditions, the Debye length was estimated as 55 nm from conductivity measurements. As a result, the gelation barrier to aggregation was lowered to less than 1 *k*_B_*T*, and the interaction potential can be thought of as purely attractive.

**Figure 5:**
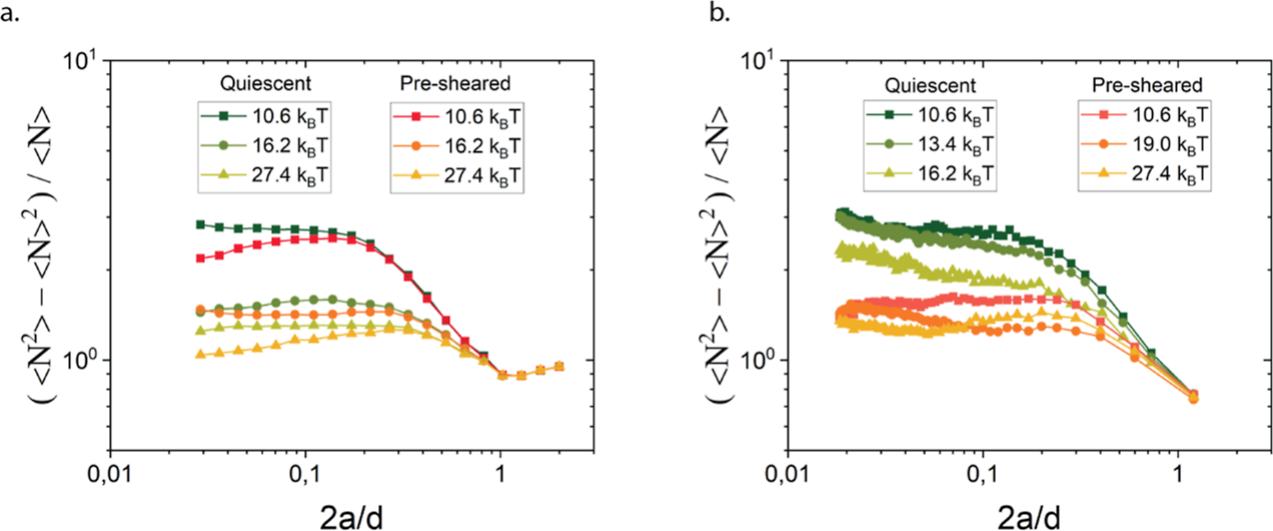
Effect of preshear and subsequent quenching versus an instantaneous quench in the absence of shear flow in simulations (a) and experiments (b).

The number density fluctuations after structural recovery upon cessation of flow at 100 s^–1^ are shown in Figure 5, alongside the same measure for gelation following salt diffusion. In the range of interparticle potentials between *U* = 5 *k*_B_*T* to *U* = 27.4 *k*_B_*T*, the presheared suspensions quickly formed a network. Within about 3 min, a terminal microstructural state was reached with no further significant rearrangements. This instantaneous quench following preshear resulted in homogeneous, yet more “stringy” networks and the spinodal structures are not found again. The qualitative shape of the number density fluctuation curves resembled those in Figure 3, but the magnitude of the fluctuations shown in Figure 5 depends on the protocol used. Smaller values of number fluctuations were obtained for structures prepared using a preshear protocol, compared to gelation protocols which involve diffusion of the salt. Although the trends in the simulations and experiments agree, with preshear reducing the magnitude of the plateau of the number density variations as a function of box size, the experiments show a stronger structural dependency on preshear. The one ingredient which is missing from the simulation is on local scale hydrodynamic effects, which could rationalize why the shear induced densification is captured in less detail.^64,65^ However, the essential ingredient which is needed to capture the essential features of the structural heterogeneity in these gels under flow are the long-range hydrodynamics, in agreement with e.g. the work by Varga et al.^66^

Interestingly, the experimental curves from the different gelation protocols are very similar, and not too dependent on the magnitude of attraction, indicating that for sufficient attraction strength (*U* ≥ 5 *k*_B_*T*) and an instantaneous quench, the resulting gel microstructure becomes only weakly dependent on the attraction strength. These results align with previous work on thermoreversible nanoemulsion gels, where quench depth dependence of heterogeneity was studied by temperature-induced gelation.^41^ For *U* = 10.6 *k*_B_*T*, the number fluctuations were smaller than those at higher depletant concentrations in quiescent gelation experiments. These results suggest that the specifics of the quenching protocol and in particular the role of hydrodynamic interactions during the quench, affect gel microstructures, even at identical attraction strengths. Both quench depth and speed relative to aggregation time play a role in determining gel network homogeneity. This agrees with observations on thermoreversible nanoemulsions, where faster quench speeds, controlled by heating rate adjustments, resulted in less heterogeneity and a 1 order of magnitude increase in storage modulus.^67^

**Figure 6:**
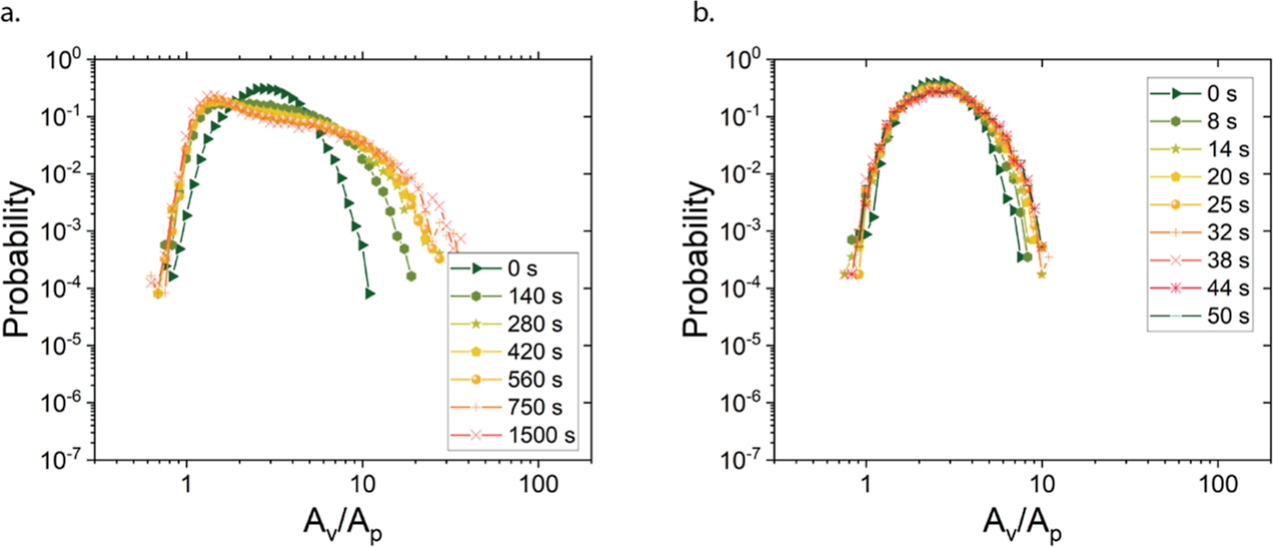
Distributions of Voronoi areas (*A*_v_) during the structural recovery following the cessation of flow at 100 s^–1^, for an interparticle potential *U* = 5 *k*_B_*T* (a) and *U* = 27.4 *k*_B_*T* (b). Results are normalized by the particle area *A*_p_.

Similar to quiescent gelation experiments with TBAC diffusion, gel formation was monitored over time after cessation of preshear flow. The Voronoi tessellation areas were tracked, resulting in the distributions shown in Figure 6. For a shallow quench at *U* = 5 *k*_B_*T*, gelation proceeded by coarsening of colloid-rich strands over long time scales, similar to the diffusion protocol. Initially homogeneous, the shear-broken aggregates formed colloid-rich strands, widening the Voronoi area distribution and increasing system heterogeneity, marked by large Voronoi cells in the gas phase and a local peak near *A*_v_/*A*_p_ ∼ 1 for tightly packed particles.

For the deepest quench at *U* = 27.4 *k*_B_*T*, the final structure was reached in less than a minute (hence the time scales in Figure 6b are much shorter), with no further changes observed in the Voronoi area distributions. The distribution remained nearly symmetrical with only slight widening and no prominent shoulder at small areas, indicating the absence of densely packed strands. The narrower, more symmetrical distribution highlights the importance of quench speed in determining gel strand size. Gel formation in this regime was diffusion-limited, trapping the system in a metastable gel state far from thermodynamic equilibrium.

**Figure 7:**
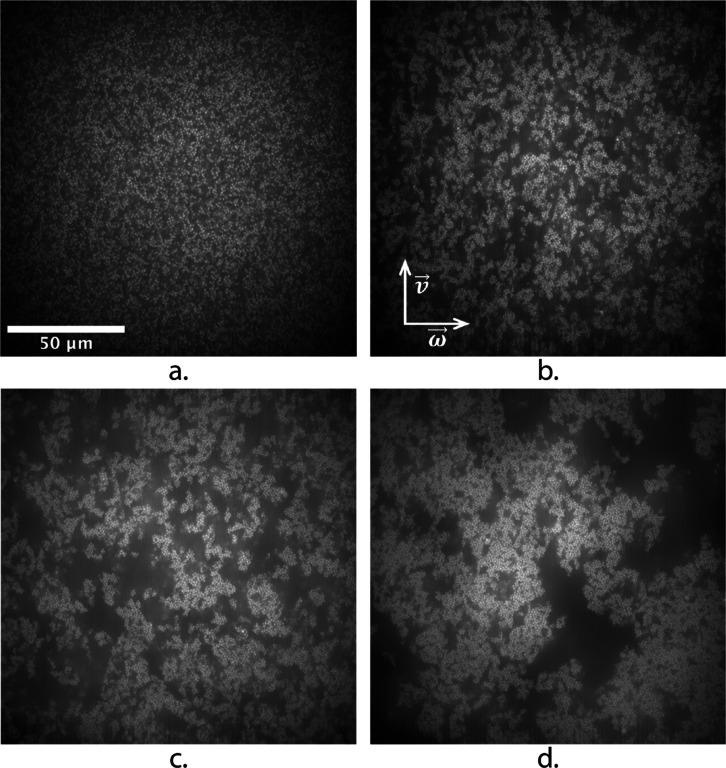
Confocal micrographs at the stagnation plane, in the steady state of simple shear of a representative depletion gel at *U* = 19 *k*_B_*T* with (a) γ̇ = 50 s^–1^, (b) γ̇ = 10 s^–1^, (c) γ̇ = 5 s^–1^, and (d) γ̇ = 1 s^–1^.

Our results are somewhat at odds with those reported by Dibble et al.,^23^ where strongly nonmonotonic variations of microstructural descriptors were found as a function of depletant concentration. In that study, samples were prepared by a mixing protocol and subsequently loaded into capillaries, without the addition of a screening salt. Gelation times and kinetics were not described in detail. The most heterogeneous reported structures show a striking resemblance with the gels sheared at intermediate flow strengths that will be discussed in the following sections (see Figure 7), as is the case for some other quiescent microstructures reported in the literature.^68^

We observe a consistent reduction in gel heterogeneity and strand size as attraction strength increases, regardless of whether a quiescent or high preshear protocol is applied. At high depletant concentrations and for instantaneous quenches, the number fluctuations become independent of the attraction strength. For these deeply quenched samples, kinetic descriptions of gelation based on rigidity^13,69^ or directed^15^ percolation are expected to be best-suited. The observed trends are in qualitative agreement with previous work on the same model system with smaller particle sizes, studied by coherent anti-Stokes Raman spectroscopy (CARS) and light scattering,^70,71^ or differential interference contrast microscopy.^72^ In those studies, premixed samples were homogenized by tumbling for long times (up to days), and then loaded for microstructural characterization.

### Structural Heterogeneity: Structures during Flow

**Figure 8:**
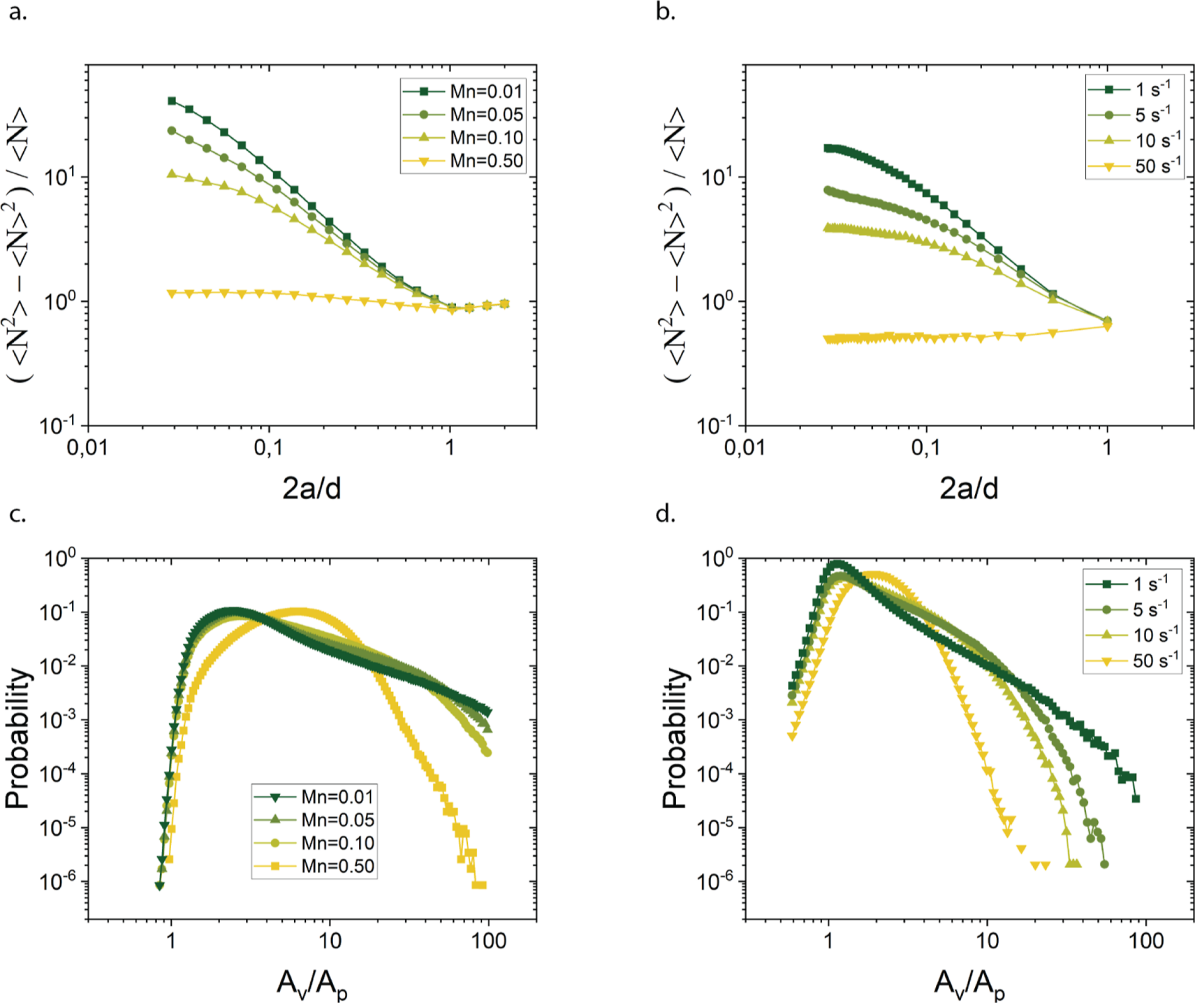
Structural characterization of gels having an attractive strength of *U* = 19 *k*_B_*T* for various nondimensional shear rates, Mason number (*Mn*), corresponding to shear rates of 2.6, 12.8, 25.6 and 127.8 s^–1^. (a,b) Number fluctuations as a function of the counting box size investigated in simulations (a) and (b) in experiments. (c,d) Distribution of normalized Voronoi tessellation areas in simulations (c) and (d) in experiments.

A preshear protocol at high Péclet number is known to be an effective method to fluidize the sample and obtain an instantaneous quench into the coexistence region, where depletion gels readily form. Shear flow at more moderate rates also dramatically affects the microstructure of the depletion gels, as shown in Figure 7 for *U* = 19 *k*_B_*T*. These confocal micrographs are representative frames of live imaging experiments at the stagnation plane (see Movies S4–S7 in Supporting Information), performed on our counter-rotating rheoconfocal setup during steady shear.^44^ A smaller magnification of 60× was used for these experiment, in order to obtain fields of view that were large enough to resolve the length scales of heterogeneity developing at moderate shear rates. Indeed, the observed microstructure under flow was strongly dependent on the applied shear rate. The striking flow-induced rearrangements shown in Figure 7 were quantified with the same structural descriptors presented above for quiescent gelation experiments. The results are summarized in Figure 8: simple shear flow was started after a preshear at 100 s^–1^ followed by a rest period of 5 min. Two minutes of shearing at the target rate were necessary to ensure a reproducible preshear and steady shear microstructures.

**Figure 9:**
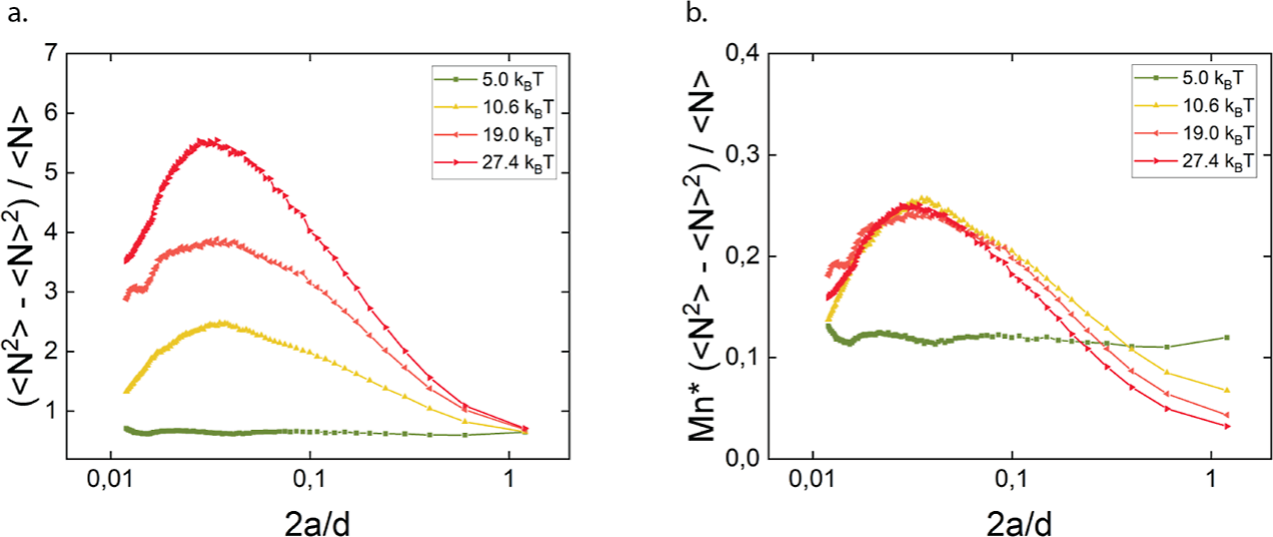
(a) Number fluctuations (⟨*N*^2^⟩ – ⟨*N*⟩^2^)/⟨*N*⟩ of depletion gels with varying attraction strength, sheared at the stagnation plane at 10 s^–1^, (b) number fluctuations at 10 s^–1^ were rescaled with the renormalized Mason number Mn*. The interparticle potential *U* is indicated in the legend.

The Péclet number compares the rate of advection due to shear to the translational Brownian relaxation of the particles. In contrast, the shear rate relative to the attraction strength is better characterized by the Mason number: . This dimensionless group was first proposed for electro-rheological fluids of non-Brownian particles^73^ and therefore does not quantify thermal motion. As a consequence, it best describes the limit of strong bonding between the particles. The same dimensionless group was called a modified Péclet number (*Pe*_dep_) in previous rheoconfocal studies on depletion gels.^64,65^ Recently, Varga and Swan^74^ proposed a renormalization of the Mason number based on the most probable rupture force f* for the interparticle bond. In this approach, the weakening of bonds caused by thermal fluctuations is explicitly taken into account, so that the resulting modified Mason number Mn* = *MnU*/(f*ξ) is expected to well characterize attractive dispersions having weaker bonds as well. Indeed, Figure 9 demonstrates a collapse of the number fluctuations for the case of γ̇ = 10 s^–1^, by multiplying the magnitude by Mn*. The curves closely overlapped at the large length scales relevant for the heterogeneity of the system. Without the renormalization by f*, the Mason number only collapsed the curves for *U* = 19 *k*_B_*T* and *U* = 27.4 *k*_B_*T*. These results suggest that starting from *U* = 10.6 *k*_B_*T*, gels were sheared in the same regime, where well-defined aggregates resulting from the interplay of shear-induced breakup and aggregation determined the compressibility of the system. However, interparticle bonds at *U* = 10.6 *k*_B_*T* were significantly affected by thermal fluctuations, whereas *U* = 19 *k*_B_*T* and *U* = 27.4 *k*_B_*T* approached the limit case of strong, athermal bonding. The magnitude of the number fluctuations remained distinct at the lowest interparticle potential *U* = 5 *k*_B_*T*, as no large aggregates and appreciable long-range heterogeneity were present in this case.

Using orthogonal superposition rheometry measurements^75^ on a strongly attractive, dilute fumed silica model thixotropic dispersion, similar trends were found in the steady shear response. There, orthogonal superposition moduli and phase angles hinted at the same mechanism of aggregate size regulation by the balance of shear-induced breakup and interparticle attraction. The gradual evolution of the microstructure as flow was increased was interpreted in terms of the Bingham number, where the shear stress is normalized by the yield stress of the studied suspension, which is proportional to the maximal attractive interparticle force. An estimation of the Mason number was not possible in that case, as the interaction strength was not known precisely.

In conclusion, shear flow at a constant and uniform shear rate within the range of intermediate Mason numbers, leads to a change in the heterogeneity of the gel. By selecting γ̇ = 10 s^–1^, we ensured that for the three highest attraction strengths the scaled Mason number remained within a heterogeneous-flow regime (*Mn* = 0.1–10). Within this regime, the data collapse demonstrates that the rescaled Mason number effectively captures the force balance controlling mesoscale structural evolution, even under flow. Although this scaling also holds for other shear rates in the same range, it deteriorates as Mason numbers approach the critical value at which the structure fluidizes. Thus, the scaling in the heterogeneous-flow regime should not be surprising; it clarifies that the rescaled Mason number indeed captures the essential physics governing the interplay between hydrodynamic forces and attractive interactions.

## Discussion

**Figure 10:**
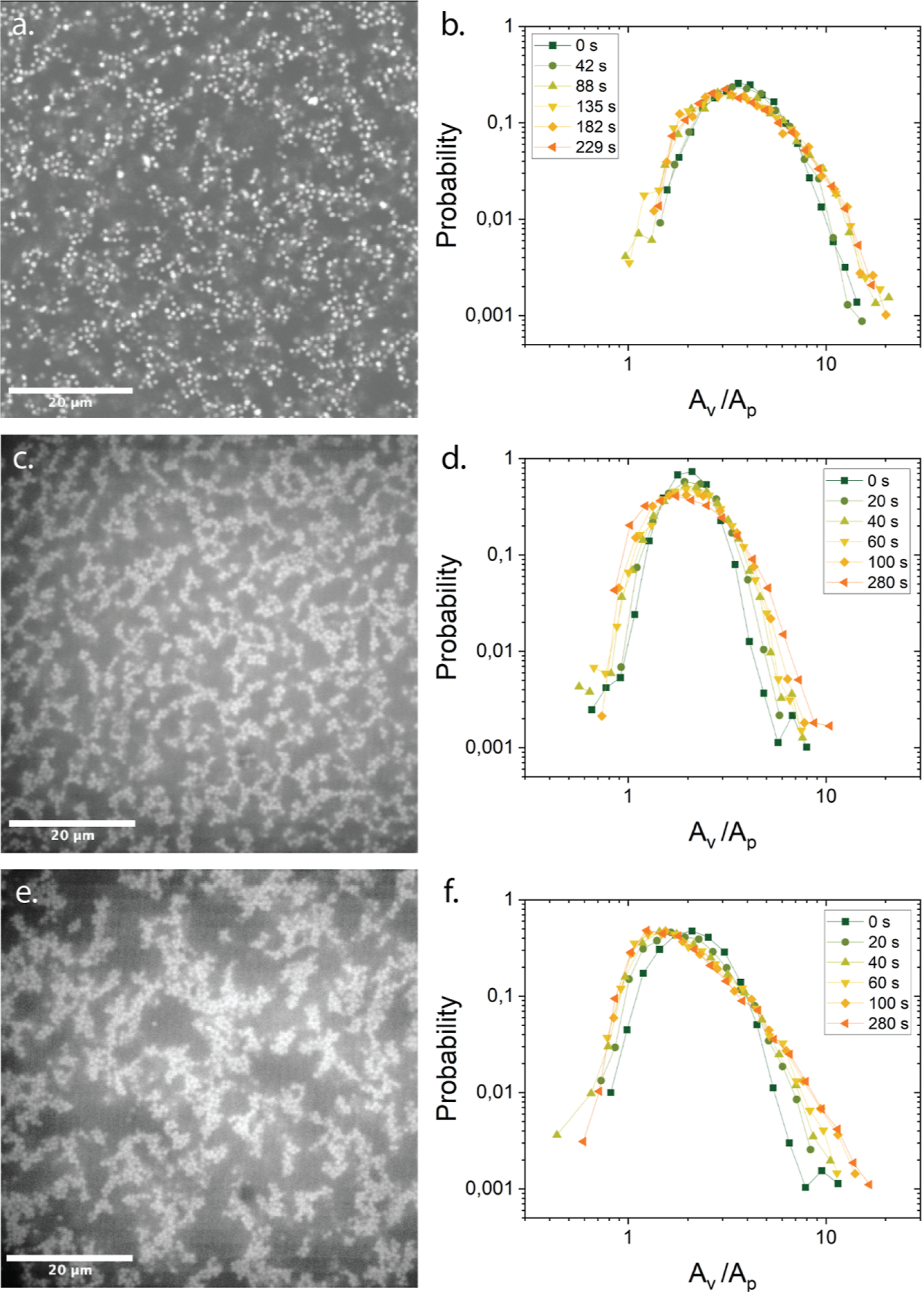
Comparison of the kinetic pathway of gelation of gels at *U* = 16.2 *k*_B_*T*. (a) Confocal micrograph of the structure induced by salt diffusion quench with the (b) time evolution of the corresponding Voronoi tessellation. (c) Confocal micrograph of the structure following preshear at a *Mn* > 10 with the (d) time evolution of the corresponding Voronoi tessellation (e) confocal micrograph of the structure following manual mixing with the (f) time evolution of the corresponding Voronoi tessellation.

The details of the quenching protocol significantly affect gel microstructures, even with identical attraction strengths. Both quench depth and speed relative to the aggregation time influence gel network homogeneity. Quiescent gelation experiments using the diffusion of TBAC salt show that gel formation can be monitored quite nicely and agree with a slow quench, with an arrested spinodal structure (see an example of this in Figure 10a,b), of which the arrest occurs earlier and earlier as the attraction strength is increased and the structure refines monotonically, as is shown in Figure 1. This contrasts with Dibble et al.,^23^ who reported nonmonotonic microstructural and heterogeneous dynamic variations with depletant concentration. Reproducible structures could also be obtained for protocols where the structure was presheared (Figure 10c,d). During flow the number density variations change, but in a way which is controlled by the renormalized Mason number. When the sample is presheared at *Mn* > 10, the structure is shear melted. Upon stopping the flow a reproducible structure develops, quite homogeneous, but different from the structures obtained by the salt induced diffusion (see Figure 10a,b versus c,d). Heterogeneous structures were only obtained when mixing the sample by hand or using a vortex mixer. Figure 10e shows a much wider distribution of voids. These qualitative changes in the micrographs in Figure 10 can be quantified through the Voronoi tessellation, where the structure which was manually mixed, shows the clearest shoulder at *A*_v_/*A*_p_ ∼ 1. This highlights a densification of the colloidal strands (Figure 10f). This shoulder is less prominent in the presheared and quiescently gelled samples, indicating a more open and stringy network structure (Figure 10b,d) We therefore suggest that the variations in shear rate and salt diffusion profiles during simple mixing result in more heterogeneous structures. Even for depletion systems at moderate strengths the kinetic pathways to gelation matter, and simple mixing protocols may under certain conditions lead to more heterogeneous structures due to spatial and temporal variations of the concentration fields of particles and solutes, and varying rates of deformation.

## Conclusion

This study provides an in-depth examination of the impact of attraction strength and shear or mixing flows on the microstructure of depletion gels with short-ranged attractions. By first employing a method of diffusing a screening organic salt into initially charge-stabilized colloid–polymer mixtures, we avoided the complicating shear history effects typical of traditional mixing protocols, thus allowing a clear study of the initial phases of gel formation. The solvent mixtures, prepared with known quantities of dissociating HBr, were carefully characterized to ensure reproducible particle charging. Our findings indicate that the resulting quiescent gel structures exhibit a consistent decrease in heterogeneity and strand size as the attraction strength increases. The initial gelation occurs at the boundary of the gas–liquid coexistence region, with deeper quenches leading to earlier arrest.

The quenching rate’s influence was demonstrated by observing gelation following the cessation of high-rate preshear, effectively creating an instantaneous quench. For sufficient attraction strengths, the microstructure was found to be essentially independent of the depletant concentration. Moreover, the number density fluctuations significantly decreased compared to the diffusion protocol, where the quench rate was determined by the diffusion time of the screening salt.

Shear flow played a critical role in restructuring the gels at all studied rates, as evidenced by live confocal imaging at a stagnation plane using a counter-rotating rheoconfocal setup. Sufficiently high shear rates (*Mn* > 10) were capable of fully fluidizing the samples, in line with reports of e.g. Petekidis and co-workers^25,26,65^ while lower shear rates led to the formation of well-defined, dense aggregates that tumbled and collided at the stagnation plane. At even lower shear rates, a transient, highly heterogeneous network with large voids and dense colloid-rich regions was observed. The number density variations in the flow regime, where aggregates formed, could be collapsed at relevant large length scales by rescaling with a modified Mason number. This rationalized the competition between shear-induced breakup and reaggregation, which dictates the aggregate size and structural heterogeneity at these rates.

The presented data set underscores the sensitivity of depletion gels to shear deformations, particularly for deeply quenched suspensions. We argue that any investigation of such model systems should incorporate a preparation protocol that avoids or mitigates previous shear history. Minimal shear deformation can lead to structural rearrangements that overshadow the effects of attraction strength, a factor often central to experimental investigations.

## Supporting Information Available

Additional experimental and characterization details including the calculated interparticle potentials, confocal micrographs of additional depletant concentration as well as selected additional information on the microstructure (PDF)
Selected movies can be found on the web site showing (i) the gelation process for the interparticle potentials of *U* = 5 *k*_B_*T*, *U* = 7.8 *k*_B_*T* and *U* = 13.4 *k*_B_*T* as well as (ii) shearing at the stagnation plane, where a gel of interparticle potential *U* = 19 *k*_B_*T* was sheared at 50, 10, 5 and 1 s^–1^ (ZIP)
