# Supplementary material for: Kinetic Pathways to Gelation and Effects of Flow-Induced Structuring in Depletion Gels
Source: Ind Eng Chem Res. 2025 Feb 12;64(8):4581–95. doi: 10.1021/acs.iecr.4c03873 (PMC11869300; doi:10.1021/acs.iecr.4c03873)
Supplement: Supplementary file 1 — ie4c03873_si_001.pdf [file ie4c03873_si_001.pdf]

Supplementary Information: Kinetic Pathways to Gelation and Effects of Flow-Induced Structuring in Depletion Gels

Gabriele Colombo [a),1] Pierre Lehéricey [a),1] Florence J. Müller [a),1] Madhu V. Majji,[2] Hanumantha Rao Vutukuri,[1,3] James W. Swan [b),2] and Jan Vermant [c)1]

[1)] *Department of Materials, ETH Zürich, CH-8093 Zürich, Switzerland*

[2)] *Department of Chemical Engineering, Massachusetts Institute of Technology, Cambridge, MA 02139, USA*

[3)] *Active Soft Matter and Bio-inspired Materials Lab, Faculty of Science and Technology, MESA+ Institute, University of Twente, 7500 AE Enschede, The Netherlands*

(Dated: 30 January 2025)

This supplementary information contains:

Number of pages: 5

Number of movies: 7 (online, captions provided below)

Number of figures: 6

[a)] these authors contributed equally

[b)] deceased

[c)] corresponding author: jan.vermant@mat.ethz.ch

Captions for the online movies

Movie S1: Gelation process at 5 $k_B T$.

Movie S2: Gelation process at 7.8 $k_B T$.

Movie S3: Gelation process at 13.4 $k_B T$.

Movie S4: Shearing of a gel of 19 $k_B T$ at a strain rate of $\dot{\gamma} = 50\ s^{-1}$.

Movie S5: Shearing of a gel of 19 $k_B T$ at a strain rate of $\dot{\gamma} = 10\ s^{-1}$.

Movie S6: Shearing of a gel of 19 $k_B T$ at a strain rate of $\dot{\gamma} = 5\ s^{-1}$.

Movie S7: Shearing of a gel of 19 $k_B T$ at a strain rate of $\dot{\gamma} = 1\ s^{-1}$.

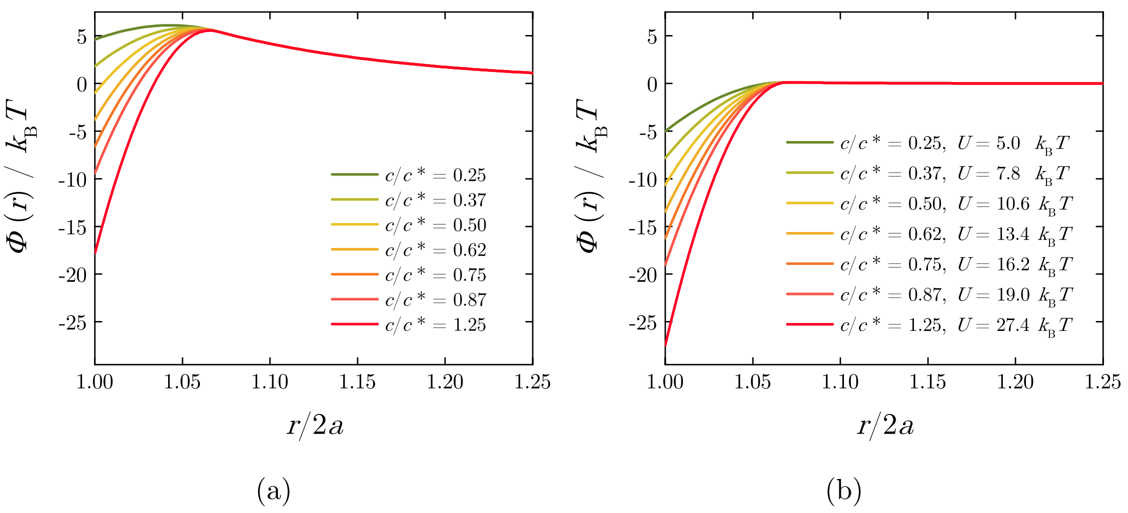

FIG. S1: Interaction potential $\Phi(r)$ estimated from the sum of an attractive, Asakura–Oosawa and a screened Coulomb interaction. The electrostatic repulsion was significant without addition of TBAC (a, $\kappa^{-1} = 150$). Addition of the screening salt lead to a purely attractive interaction potential (b, $\kappa^{-1} = 55$). The depletant concentration is indicated in the legend, alongside the contact attraction strength U in (b).

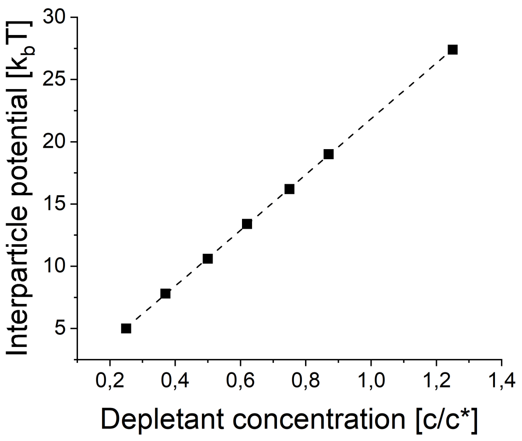

FIG. S2: Relation between depletant concentration and interparticle potential used to compare simulations and experimental data.

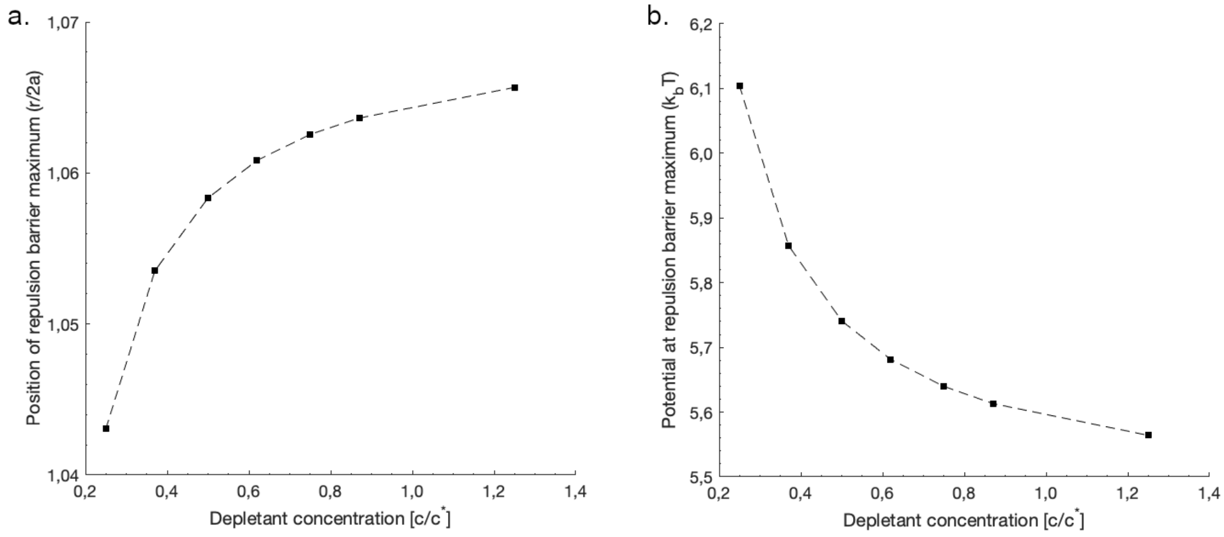

FIG. S3: Evolution of the position (a) and the height (b) of the barrier to gelation in the sums of the Asakura Oosawa and Yukawa potentials from FIG. S1a.

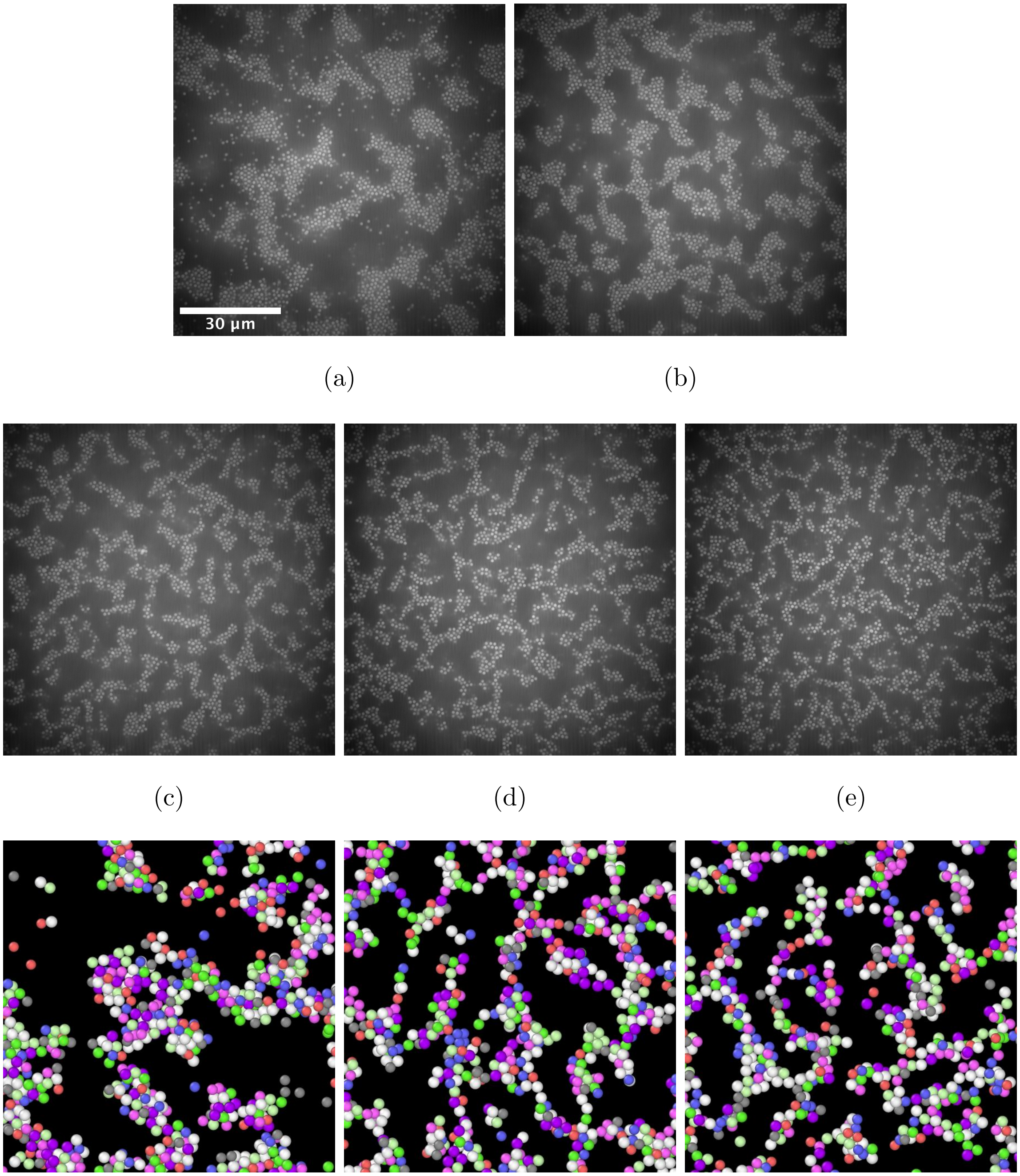

(a) (b) (c) (d) (e) (f) (g) (h)

FIG. S4: Representative confocal micrographs of depletion gels obtained by the salt screening quiescent gelation protocol, as a function of the attraction strength. (a) $c/c^* = 0.25$ ($U = 5\ k_BT$), (b) $c/c^* = 0.37$ ($U = 7.8\ k_BT$), (c) $c/c^* = 0.5$ ($U = 10.6\ k_BT$), (d) $c/c^* = 0.62$ ($U = 13.4\ k_BT$), (e) $c/c^* = 0.75$ ($U = 16.2\ k_BT$). Gel microstructure predicted from simulation (f) $c/c^* = 0.5$ ($U = 10.6\ k_BT$), (g) $c/c^* = 0.75$ ($U = 16.2\ k_BT$), (h) $c/c^* = 1.25$ ($U = 27.4\ k_BT$).

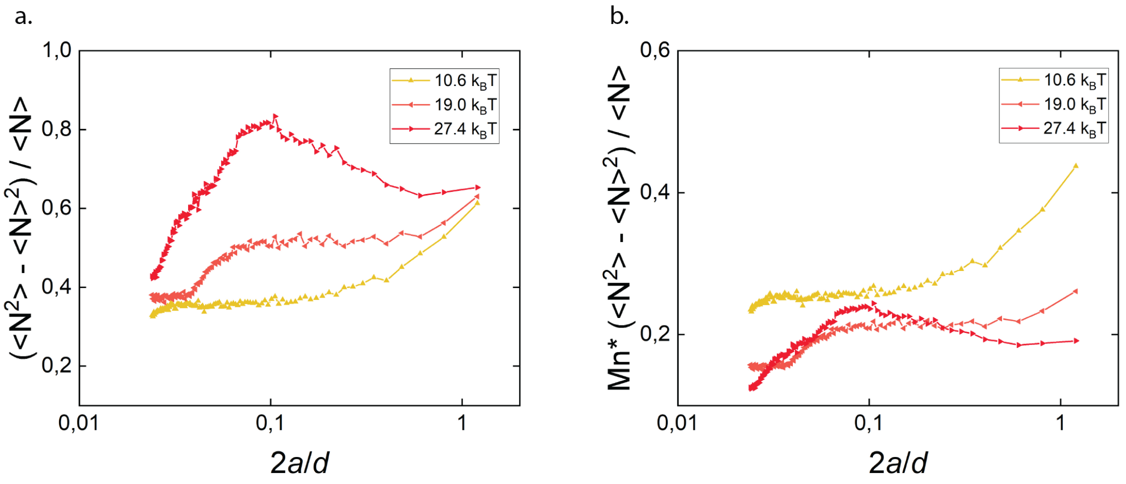

FIG. S5: a. Number fluctuations $\left(\langle N^2\rangle - \langle N\rangle^2\right)/\langle N\rangle$ of depletion gels with varying attraction strength, sheared at the stagnation plane at 50 s^{-1}, b. Number fluctuations at 50 s^{-1} were rescaled with the renormalized Mason number Mn*. The interparticle potential U is indicated in the legend.

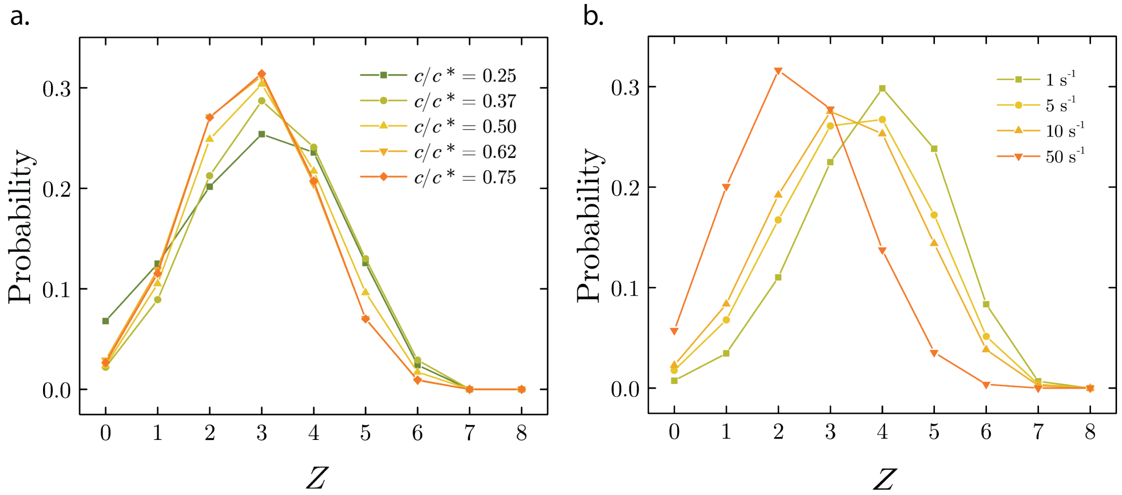

FIG. S6: Distribution of the coordination number Z as a function of a. interparticle attraction strength during quiescent gelation and b. Shear rate for $c/c^* = 0.75$ ($U = 16.2\ k_BT$)
